# One-stage surgical management for advanced dilated cardiomyopathy combined with aortic sinus aneurysm: a case report

**DOI:** 10.3389/fcvm.2025.1600757

**Published:** 2025-08-22

**Authors:** Wenliu Xu, Chaojie Wang, Zengxiao Zou, Ge Wang, Songtao Tan, Xiaoping Fan

**Affiliations:** Department of Cardiovascular Surgery, Guangdong Provincial Hospital of Chinese Medicine, The Second Affiliated Hospital of Guangzhou University of Chinese Medicine, Guangzhou, China

**Keywords:** left ventricular assist device implantation, aortic surgery, end-stage dilated cardiomyopathy, aortic sinus aneurysm, case report

## Abstract

**Introduction:**

Left ventricular assist device (LVAD) implantation is a highly effective procedure for the management of selected advanced heart failure patients, prolonging patient life and improving quality. Additional cardiac pathologies, especially valvular regurgitation or coronary heart disease, are common in LVAD recipients, whereas reports on the surgical management of heart failure combined with aortic disease are rare.

**Case presentation:**

We present a case of a 60-year-old patient with an aortic sinus aneurysm, aortic regurgitation, and end-stage heart failure. LVAD implantation was performed concomitantly with the Bentall procedure and Cabrol shunt technique. The patient was discharged uneventfully on postoperative day 26. This suggests that combining LVAD implantation with additional cardiovascular procedures could be an alternative strategy for patients with complex heart failure conditions.

**Conclusion:**

LVAD implantation combined with additional aortic surgery can be a feasible alternative with acceptable risk, especially for patients who have elected to pursue LVAD as destination therapy. Successful outcomes require adequate preoperative evaluation, experienced cardiac surgeons, and close postoperative care.

## 1 Introduction

Left ventricular assist device (LVAD) implantation is a highly effective procedure for the management of selected advanced heart failure patients, prolonging patient life and improving quality. Additional cardiac pathologies, especially valvular regurgitation or coronary heart disease, are common in LVAD recipients; however, reports on the surgical management of heart failure combined with aortic disease are rare (1–4). Here, we report a case of end-stage dilated cardiomyopathy with aortic sinus aneurysm and aortic regurgitation treated with LVAD implantation combined with the Bentall procedure and Cabrol shunt technique.

## 2 Case report

### 2.1 Medical history

**Figure 1:**
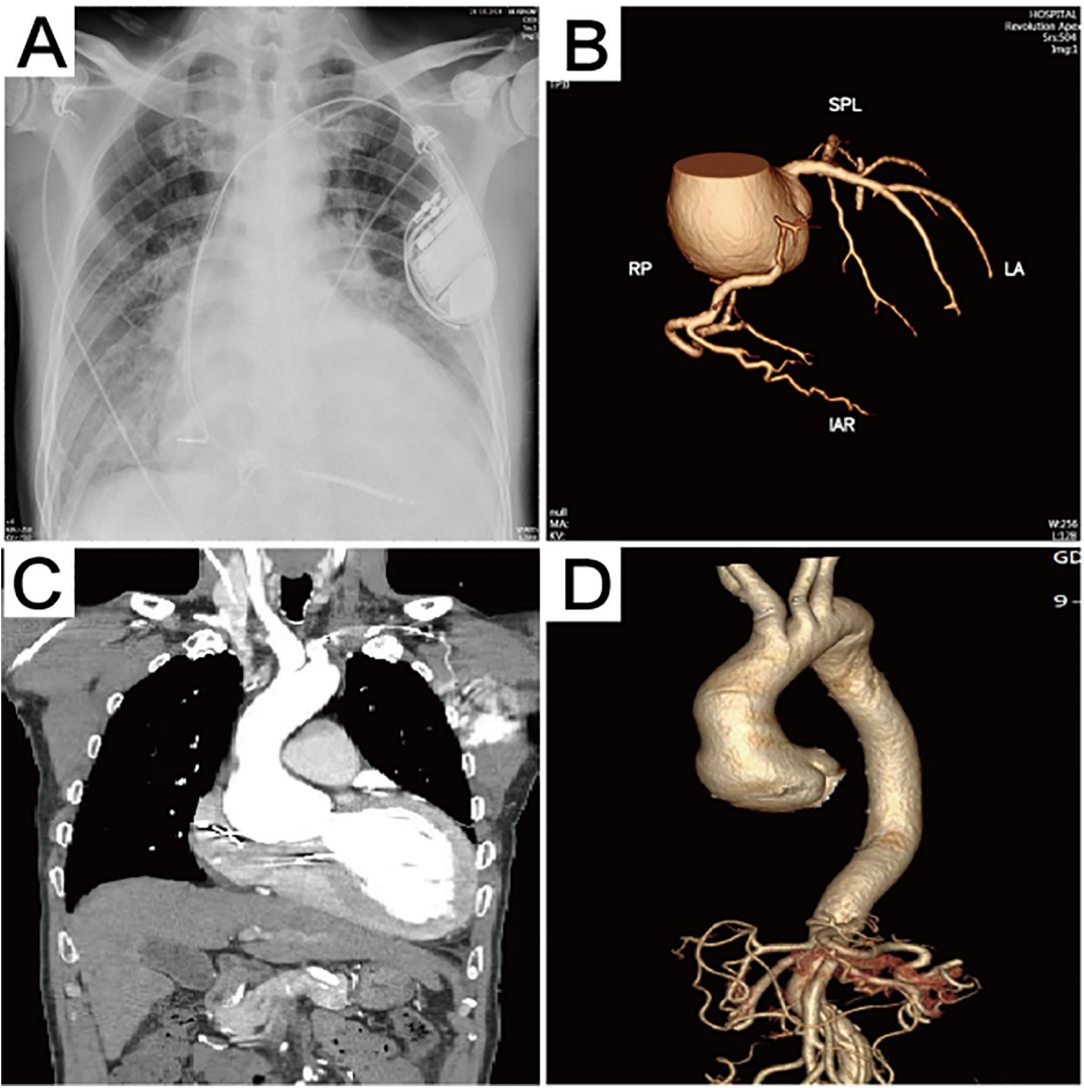
Preoperative imaging data. **(A)** The chest x-ray revealed the presence of pulmonary edema. **(B)** Computed tomography 3D reconstruction of the coronary arteries. **(C)** Preoperative computed tomography scan showing aortic sinus aneurysm, with the maximum aortic root diameter measuring 53.3 mm. **(D)** Computed tomography 3D reconstruction of the aorta.

A 60-year-old male patient (weight 60 kg, height 1.70 m, body surface area 1.72 m^2^) was admitted with a 4-year history of chest tightness and shortness of breath, which had aggravated for 1 month. Four years ago, the patient presented with chest tightness, palpitations, and shortness of breath, which were relieved by rest. The patient did not undergo further diagnostic evaluation or treatment at that time and continued to experience recurrent episodes. In 2023, an implantable cardioverter-defibrillator was implanted due to frequent ventricular premature beats, and the patient was maintained on long-term oral medication, including sacubitril/valsartan, dapagliflozin, bisoprolol, vericiguat, torasemide, and spironolactone. One month ago, these symptoms had worsened and were not relieved by resting. The patient presented with lower extremity edema and fatigue and was observed to sit upright to facilitate breathing. He was subsequently hospitalized in the hospital’s cardiovascular department. The N-terminal pro-brain natriuretic peptide (NT-proBNP) level was 17,245 ng/L, and high-sensitive cardiac troponin (hs-cTnT) level was 0.074 µg/L. Chest x-ray showed pulmonary edema (Figure 1A). He received treatment for heart failure, including cardioprotective theraphy, diuresis to reduce the cardiac load, and other symptomatic treatments. His past medical history was unexceptional.

### 2.2 Investigation

Echocardiography after admission revealed dilated cardiomyopathy, severe aortic regurgitation, mild-to-moderate mitral regurgitation, moderate tricuspid regurgitation, reduced left ventricular systolic function, and general reduction in ventricular wall motion, with an ejection fraction of 22% and a left ventricular end diastolic diameter (LVEDD) of 80 mm. Coronary computed tomographic angiography (CTA) suggested the presence of varying degrees of mild stenosis in all coronary arteries, with no significant lesions identified (Figure 1B). Aortic CTA suggested an aortic root aneurysm, with a maximum aortic diameter of up to 53 mm (Figures 1C,D). Right heart catheterization showed a central venous pressure (CVP) of 3 mmHg, mean pulmonary artery pressure of 29 mmHg, and a total pulmonary resistance of 1.35 Wood units.

### 2.3 Management

The patient was admitted with a more serious presentation of the disease. Chest radiographs indicated worsening bilateral pulmonary exudates, and his blood pressure was found to be low. A moderate-to-medium dose of dopamine was administered to assist the heart in perfusing the body. The INTERMACS score was 3, low risk was indicated by a score of 2 points on the EUROMACS-RHF score, and the EuroSCORE II was 15.35. Following a period of medical management, heart failure symptoms were notably reduced, and his lung condition showed significant improvement. The patient met the criteria for a heart transplant, and our initial recommendation was for them to wait for the procedure. Generally, it is not advisable to combine complex procedures with LVAD implantation. However, due to the shortage of heart donors in China, the patient was at high risk of dying while waiting for a transplant. Furthermore, the patient had experienced repeated heart failure and had been hospitalized several times for treatment. Our team discussed treatment options with the relatives several times, and they expressed their desire for the surgery to be performed as soon as possible. Based on the patient's and their family's decision, our team decided to perform LVAD implantation concomitantly with the Bentall procedure for the patient.

**Figure 2:**
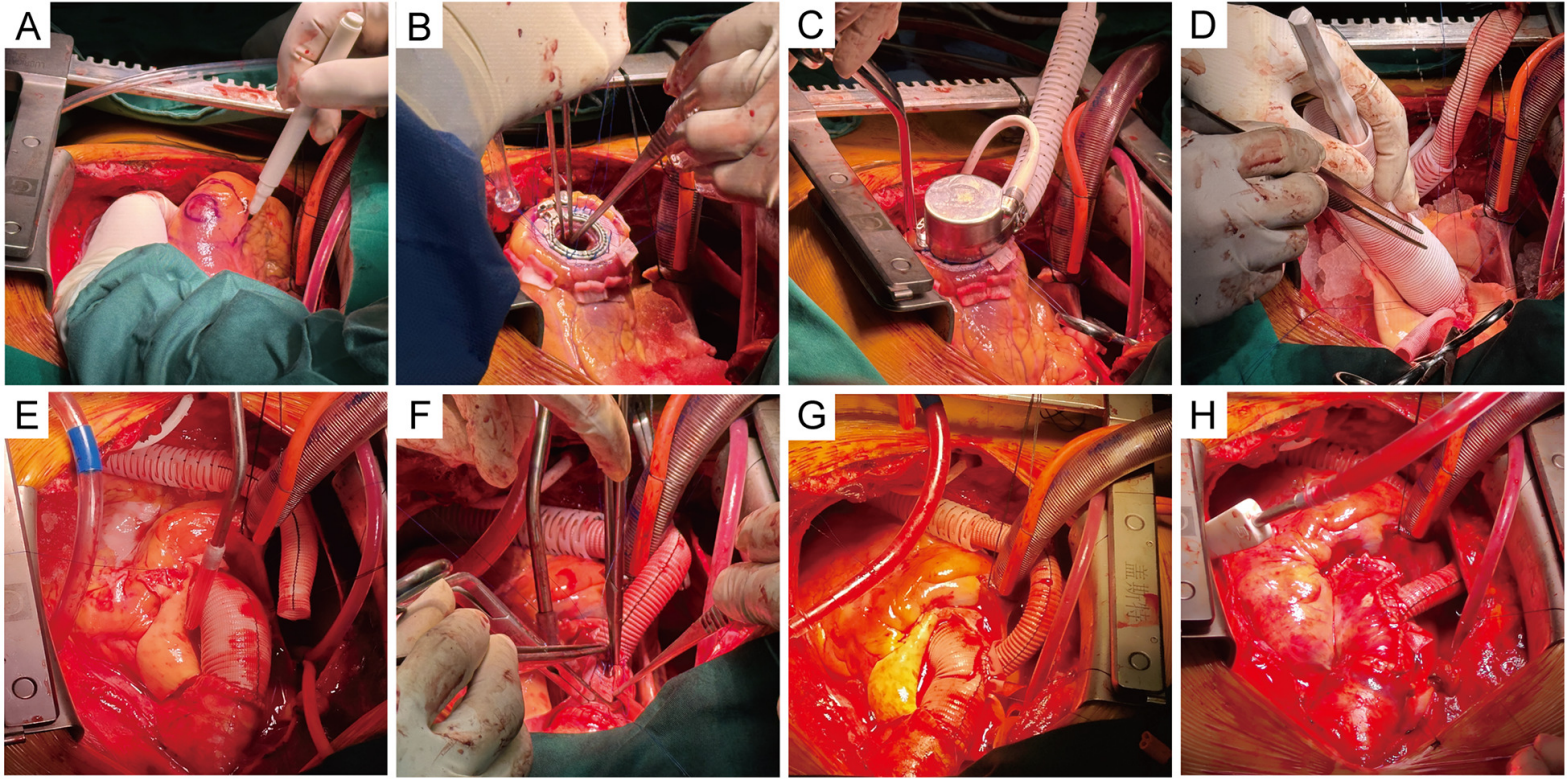
Intraoperative procedure. **(A)** The placement of the suture ring was selected and marked at the apex. **(B)** The LVAD ring was sewn on the marked region of the apex with Teflon-pledged sutures, and a 4-0 running Prolene suture was reinforced. The excess tissue in the ventricles was removed. **(C)** The inflow cannula was placed into the left ventricle and secured. **(D)** A separate 10-mm-diameter Dacron graft was anastomosed end-to-end to the left coronary. The composite conduit was sewn into place at the aortic annulus. **(E)** The anastomosis of the right and left coronary orifices was completed. **(F)** The latter conduit was anastomosed to the distal end of the aorta. **(G)** The cuff was sewn around the anastomosis on the ascending graft. The outflow graft was anastomosed end-to-side at the cuff on the ascending graft. **(H)** The ascending graft was wrapped using artificial pericardium and autologous vessel wall, and the Cabrol shunt technique was performed, showing the final surgical outcome.

After achieving general anesthesia, the right femoral artery was exposed. Subsequently, a median sternotomy was performed. Transesophageal echocardiography was used to identify the appropriate placement of the apex region. The cardiopulmonary bypass (CPB) was established by two-stage cannulation of the right atrium and femoral artery. A left ventricular drain was implanted through the right upper pulmonary vein. The ascending aorta was cross-clamped, and then the del Nido solution was perfused via the coronary orifice. The apex of the heart was elevated and exposed. The LVAD ring was then sewn on the marked region of the apex with teflon-pledged sutures (Figure 2A). The center of the sewing ring was incised using a punch. The left ventricle was then examined, after which thrombus and excess tissue were removed (Figure 2B). Thereafter, the inflow cannula was placed into the left ventricle. The outflow vascular graft was secured at its distal end (Figure 2C). The driveline was tunneled within the rectus muscle sheath.

The Bentall procedure was initiated. After excision of the aortic valve, an appropriately sized graft was selected, and then the bioprosthesis was sutured to the proximal end of the prosthetic graft to form a composite conduit. A separate 10-mm-diameter Dacron graft was trimmed to the appropriate length and anastomosed end-to-end to the left coronary orifice with a running 5-0 Prolene suture. The composite conduit was sewn into place at the aortic annulus with continuous 4-0 Prolene sutures (Figure 2D). The tissue around the right coronary orifice was released, and the right coronary artery was anastomosed end-to-side to the aortic graft directly (Figure 2E). Next, the latter conduit was anastomosed end-to-end to the distal of the aorta (Figure 2F). A partial cross-clamp was placed on the ascending aortic graft. The cuff was sewn around the anastomosis on the ascending graft using a continuous 4-0 Prolene suture. The outflow graft was cut obliquely, and then anastomosed end-to-side at the cuff with a running 4-0 Prolene suture (Figure 2G). The Cabrol shunt technique was performed to collect the bleeding from the perigraft space to the right atrium using artificial pericardium and the remaining vessel wall (Figure 2H). Rewarming and decannulation are then performed in a routine manner. While the LVAD achieved a pump ﬂow of 2.78 L/min at 2,546 rpm, the patient was weaned from the CPB. The total CPB time was 332 min, and the cross-clamp time was 225 min. The patient was transferred to the ICU.

**Figure 3:**
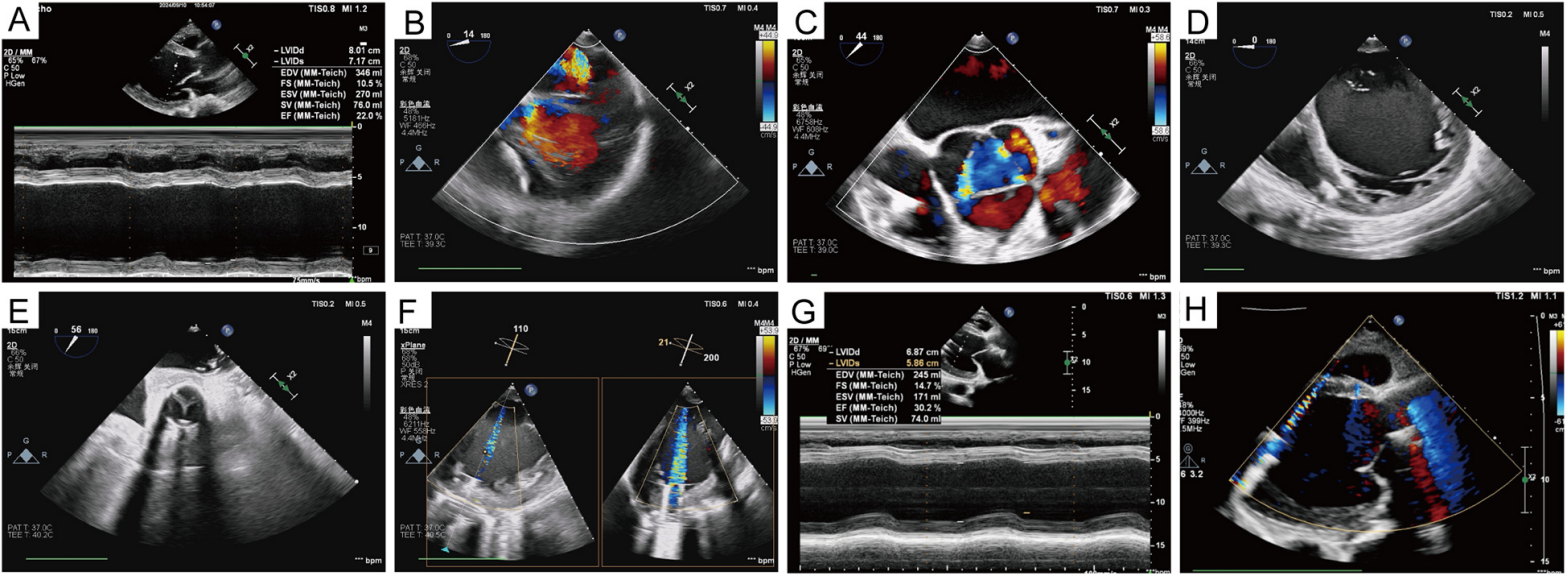
Echocardiographic data. **(A)** Preoperative ejection fraction and left ventricular diameter. **(B–D)** Mitral, aortic valve regurgitation, and left ventricular volume before left ventricular assist device implantation. **(E)** Opening and closing of the prosthetic valve. **(F)** The relationship between the inflow cannula of the left ventricular assist device and the mitral orifice and the ventricular septum was also demonstrated. **(G,H)** Left ventricular volume, as well as valve regurgitation, were significantly improved 1 year after the surgery.

**Figure 4:**
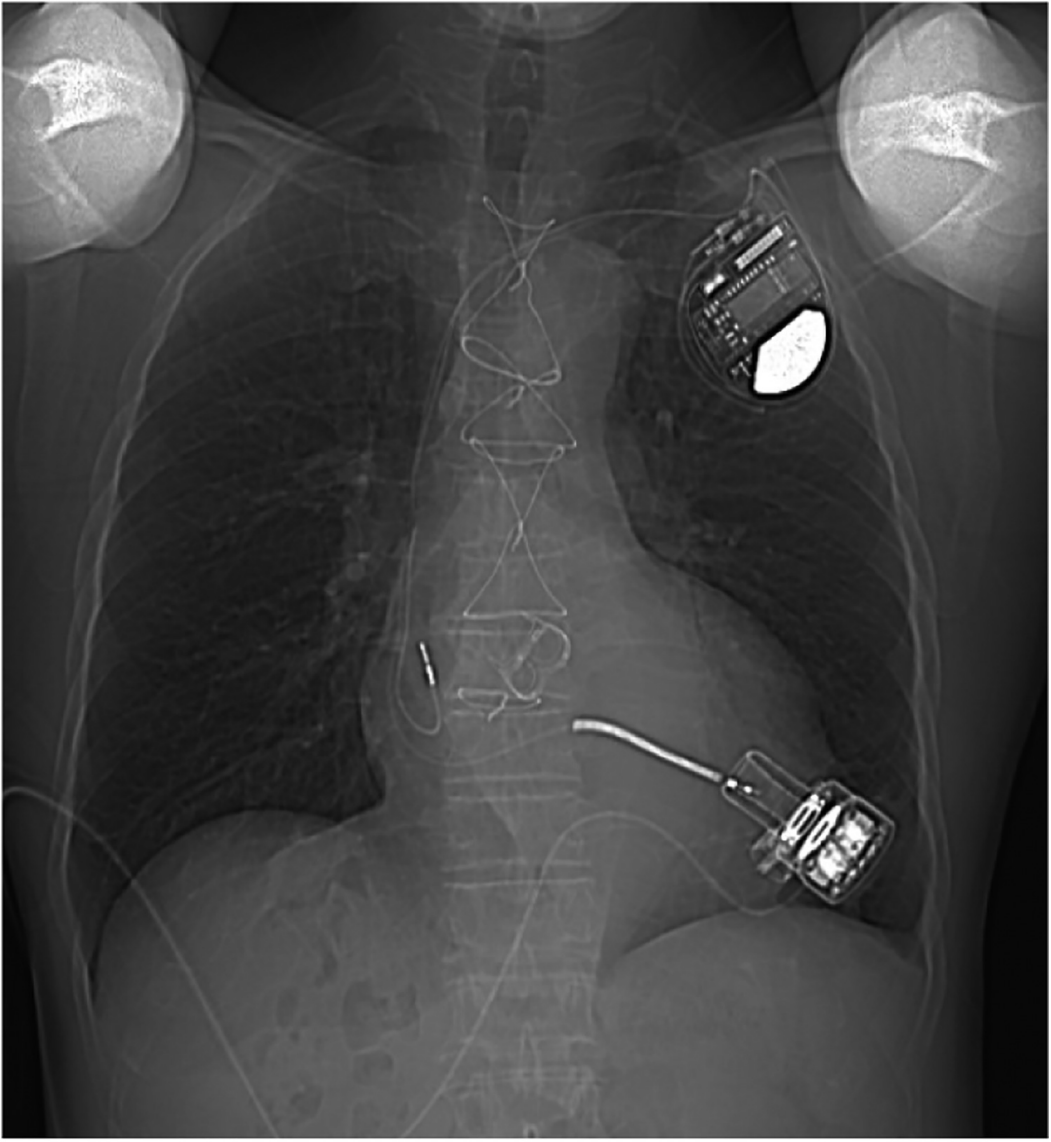
A chest x-ray was performed 1 year after discharge.

The patient exhibited oliguria on the first postoperative day (POD), accompanied by a rise in renal function index. The patient was placed on bedside continuous renal replacement therapy (CRRT). The patient's urine returned to a normal level on POD4. The patient was extubated on POD4. The patient had more pleural fluid postoperatively, which was deemed to be associated with exudate from the artificial vessel and the extensive surgical trauma. The drainage levels decreased, and the chest tube was subsequently removed on POD19. Heparin was administered, and the activated clotting time (ACT) was maintained at 160–200 s. The patient's warfarin was initiated on POD6, trying to maintain the international normalized ratio (INR) at 1.8–2.5. At 2 weeks postoperatively, the patient was able to ambulate and move without assistance. He was discharged on POD26 without any adverse events, while the LVAD achieved a pump ﬂow of 3.08 L/min at 2,846 rpm. One year after the surgery, echocardiography revealed normal prosthesis function and reduced valve regurgitation, with an ejection fraction of 30% and an LVEDD of 68 mm (Figure 3). The chest x-ray taken 1 year after the patient was discharged from the hospital is shown in Figure 4.

## 3 Discussion

A growing public health concern, heart failure is becoming increasingly prevalent among younger populations. The growing burden of heart failure in young people, which now accounts for 1%–3% of all cases, is a subset where one would consider LVAD implantation in preference to other alternatives (5). In comparison to heart transplantation, the benefit of LVADs is that patients do not need to wait for a donor and can use them immediately, whereas heart transplantation carries a high risk of death during the waiting period. After considering the patient's severe heart failure symptoms and the patient's family's wishes, which included some reservations about the long-term use of immunosuppressive drugs after transplantation and the high lifetime medication costs, our team chose this surgical procedure. The surgical strategy was developed to provide the patient with a chance to survive and recover well, regain long-term quality of life with LVAD support, and undergo heart transplantation in the future.

Certain prerequisites are necessary to assess the feasibility of surgery. Previous literature (1–4) has reported on this type of LVAD combined with aortic lesion treatment surgery, and the details are compared in Table 1. Preoperative cardiac echocardiography and right heart catheterization revealed satisfactory right heart reserve function. Although the patient presented with obvious heart failure upon admission, comprehensive treatment with cardiac stimulants, diuretics, and anti-heart failure drugs resulted in a good recovery of all the patient's organs, including the lungs, liver, and kidneys. Prompt intervention was required for severe aortic valve regurgitation combined with aortic root dilation. These complications may include rupture, dissection, and the formation of a pseudoaneurysm and can lead to life-threatening aortic events if left untreated. Our collaborative cardiac team includes cardiac surgeons, cardiologists, anesthetists, echocardiography specialists, and other experts. The cardiac surgeon is an expert in cardiac and aortic surgery.

**Table 1:** Comparison of details with those in recent similar literature.

Study	Age, y	Gender	End-stage heart failure	Aortic valve insufficiency	Sinuses of Valsalva	Ascending aorta	Coronary heart disease	LVAD	Concomitant procedure	Survive
Netuka et al. (1)	55	Male	Ischemic cardiomyopathy	No	No	Aortic dissection	Yes	HeartMate II	Hemi-arch technique	Yes
Takeda et al. (4)	38	Male	Dilated cardiomyopathy	No	Saccular aneurysm	Several saccular aneurysms	No	HeartWare HVAD	Sinus of Valsalva repair, ascending aortic replacement	Yes
Gode et al. (3)	46	Male	Dilated cardiomyopathy	Severe	Aortic root dilatation	Ascending aortic aneurysm	No	HeartMate 3	Bentall, ascending aortic replacement	Yes
Zhao et al. (2)	62	Male	Dilated cardiomyopathy	Severe	Aortic root dilatation	Ascending aortic aneurysm	Yes	CH-VAD	Bentall, ascending aortic replacement, CABG, Cabrol shunt	Yes
This study (2024)	60	Male	Dilated cardiomyopathy	Severe	Aortic root dilatation	Ascending aortic aneurysm	No	CoreHeart 6	Bentall (Cabrol technique), ascending aortic replacement, Cabrol shunt	Yes

CABG, coronary artery bypass grafting.

In this case, we did not address the mitral valve and tricuspid valve lesions. On the one hand, dealing with the other valves would have prolonged the patient's cross-clamp time and operative time, exposing him to higher risks. On the other hand, it was possible to reduce left ventricular volume while mitigating mitral regurgitation after LVAD implantation (6). Current guidelines state that routine repair or replacement for mitral regurgitation is not recommended (7). Some literature report that tricuspid valve intervention at the time of LVAD implantation has no significant impact on clinical outcomes (8, 9). The problem of aortic regurgitation becomes an increasing concern over time after LVAD implantation (10). Transcatheter aortic valve replacement (TAVR) would be an alternative approach, if the patient develops severe aortic regurgitation with LVAD support in the future. In this case, we chose a restrictive expandable bioprosthesis. In the event of valve-in-valve treatment for bioprosthesis degeneration in the future, an interventional balloon can be used to expand the annulus of this prosthesis to further increase its diameter and orifice area to accommodate a larger-sized prosthesis implant for better results.

In dealing with the left coronary orifice, the Cabrol procedure of the aortic root was performed, offering a tension-free anastomosis, which attached the composite valve graft to the coronary orifice with a separate 10 mm Dacron graft to prevent pseudoaneurysms (11). The right coronary orifice was anastomosed directly to the aortic graft to simplify the management. Sewing a circle of prosthetic material around the anastomosis as a cuff, between the outflow graft of the pump and the composite graft, was convenient to wrap the ascending graft completely using prosthetic material or autologous aortic wall. This could not only be extremely effective at controlling bleeding from suture lines but could also reduce postoperative chest drainage to some degree.

Perioperative care also plays an important role in this case. The patient's postoperative oliguria and progressive increase in urea nitrogen and creatinine levels, combined with low cardiac output and cardiac index, led us to suspect a decline in cardiac function due to excessive volume overload; therefore, we administered continuous bedside dialysis. As the volume load decreased, the patient's cardiac output and cardiac index gradually increased, and the patient's urine gradually returned to normal levels. After the operation, daily echocardiograms were performed to monitor the left and right heart volumes and the position of the interventricular septum, which informed the next step in treatment. The patient's postoperative pleural fluid drainage was excessive, which was thought to be related to exudation from the vascular graft and surgical wound, and an amount of plasma albumin was required to provide adequate colloid osmotic pressure.

In conclusion, our case shows that LVAD implantation combined with additional aortic surgery can be a feasible alternative with acceptable risk, especially for patients who have elected to pursue LVAD as destination therapy. Concomitantly, however, the disadvantages of this procedure must also be acknowledged. The complexity of the surgery is directly associated with increased duration of cardiopulmonary bypass and surgery and consequently heightened likelihood of postoperative complications and risks. There is a need to follow up on the long-term outcomes for the patient.

## Data Availability

The original contributions presented in the study are included in the article/Supplementary Material; further inquiries can be directed to the corresponding authors.

## Supplementary material

Supplementary Video S1 Video recording of intraoperative transesophageal echocardiography.
